# Thymosin Beta-4 Modulates Cardiac Remodeling by Regulating ROCK1 Expression in Adult Mammals

**DOI:** 10.3390/ijms26094131

**Published:** 2025-04-26

**Authors:** Klaudia Maar, Jeffrey E. Thatcher, Egor Karpov, Szilard Rendeki, Ferenc Gallyas, Ildiko Bock-Marquette

**Affiliations:** 1 Department of Biochemistry and Medical Chemistry, University of Pecs Medical School, 7624 Pecs, Hungary; 2 Department of Cardiovascular and Thoracic Surgery, University of Texas Southwestern Medical Center, Dallas, TX 75390, USA; 3 Department of Anesthesiology and Intensive Therapy, University of Pecs Medical School, 7624 Pecs, Hungary; 4 Medical Skills Education and Innovation Centre, University of Pecs Medical School, 7624 Pecs, Hungary; 5 Department of Molecular Biology, University of Texas Southwestern Medical Center, Dallas, TX 75390, USA

**Keywords:** thymosin beta-4, cardiac regeneration, ROCK1, miR139-5p

## Abstract

Although a myocardial infarction occurs roughly every minute in the U.S. alone, medical research has yet to unlock the key to fully enabling post-hypoxic myocardial regeneration. Thymosin beta-4 (TB4), a short, secreted peptide, was shown to possess a beneficial impact regarding myocardial cell survival, coronary re-growth and progenitor cell activation following myocardial infarction in adult mammals. It equally reduces scarring, however, the precise mechanisms through which the peptide assists this phenomenon have not been properly elucidated. Accordingly, the primary aim of our study was to identify novel molecular contributors responsible for the positive impact of TB4 during the remodeling processes of the infarcted heart. We performed miRNA profiling on adult mice hearts following permanent coronary ligation with or without systemic TB4 injection and searched for targets and novel mechanisms through which TB4 may mitigate pathological scarring in the heart. Our results revealed a significant increase in miR139-5p expression and identified ROCK1 as a potential target protein aligned. Real-time PCR, Western blot and immunostaining on adult mouse hearts and human cardiac cells revealed the peptide indirectly or directly modulates ROCK1 protein levels both in vivo and in vitro. We equally discovered TB4 may reverse or inhibit fibroblast/myofibroblast transformation and the potential downstream mechanisms by which TB4 alters cellular responses through ROCK1 are cell type specific. Given the beneficial effects of ROCK1 inhibition in various cardiac pathologies, we propose a potential utilization for TB4 as a ROCK1 inhibitor in the future.

## 1. Introduction

Cardiovascular disease (CVD) encompasses a range of conditions affecting the heart and blood vessels. It is a leading global health concern, contributing to significant morbidity and mortality. The primary types of heart disease include coronary artery disease, heart failure (HF), arrhythmias and valvular malfunctions. Among those, coronary heart disease (CHD) is the number one global killer accountable for nearly sixteen percent of total deaths of all causes [1]. In the United States alone, about 15.5 million people are affected and it is estimated every forty seconds, one individual succumbs to complications regarding myocardial infarction (MI) [2]. Furthermore, CHD is the most common cause of sudden death in those under the age of forty, negatively impacting society [3]. Therefore, it is essential to highlight the demand for prevention and timely effective therapy for survivors of CHD events.

Among all etiologies, the most important and common background for CHD is atherosclerotic stenosis-induced hypoxia with subsequent inflammation in the coronary artery [4]. Following infarction, the heart attempts to mend the damage via a complex process known as remodeling. Cardiac remodeling is a physiological and pathological condition with a vast number of molecular, cellular and interstitial alterations leading towards heart failure [5]. Clinical manifestations are contingent upon alterations in size, shape and function, triggered by diverse changes in gene expression [5]. Namely, due to hemodynamic and metabolic stress within the first few hours, the fetal gene program is considered predominantly active, while the postnatal gene program becomes silenced [6,7]. The following phase is considered as an adaptive process to maintain the heart function and compensate for acute phase cardiac injuries. Regrettably, it results in a maladaptive process, which is linked to HF progression [5]. Due to compensation by cellular and molecular components of the heart, fibroblasts become activated, which primarily stimulates collagen synthesis [5]. Consequentially, the necrotic area becomes fibrotic and loses its contractile function [5,8], thus, the pumping capacity of the heart diminishes. The degree of this decline is dependent upon a variety of factors, including the size of the fibrotic area [5]. In addition to scarring, the surviving myocytes change their shapes and become hypertrophied [5], thus, the thickness of the ventricular wall gradually increases to maintain contraction power [5]. Nevertheless, at a certain point, all will lead to chronic heart failure (CHF) [9], equally referred to as ischemic cardiomyopathy [8].

The aforementioned morphological alterations of remodeling are initiated and supported by numerous molecular interchanges. Within this intricate signaling network, the specific processes include myocyte loss, cardiac hypertrophy, disruption of extracellular matrix homeostasis, fibrosis, impaired autophagy, metabolic abnormalities and mitochondrial dysfunction [10]. To successfully subdue these pathological processes, it is essential to precisely identify the elements of the disease pathology which must be potentially targeted to achieve clinical and functional improvement. Undoubtedly, lowering the production of the rigid connective scar is one of the major goals and a primary target of the battle.

The importance of cellular microenvironment in relation to cell transformation and behavior became increasingly apparent and supported by recent investigations. Fibroblasts are extremely sensitive to physical stimuli such as tissue rigidity, shear forces or strains [11,12]. They engage with the extracellular environment through a cohesive structural complex, which includes the extracellular matrix (ECM), integrin-associated focal adhesions and actin cytoskeleton, all responsible for integrating environmental inputs into consequential transcriptional events. Clearly, the arrangements made in the actin cytoskeletal system are critical regarding cellular behavior and transformations and thus, myofibroblast differentiation [13]. RhoGTPases are recognized to reorganize actin cytoskeleton into stress fibers that, in a myofibroblast, contain primarily smooth muscle isoforms [14]. The most investigated RhoGTPases, Rho, Rac and CDC42 are activated by profibrotic receptor complexes and regulate effector proteins, which modulate the polymerization equilibrium of G-actin and F-actin in the cytoplasm. Such regulatory downstream targets are the Rho-associated kinases (ROCK1 and ROCK2) and the diaphanous family of formins (mDia), which are both required for polymerization and interconnection of stress fibers. Notably, pharmacologic inhibition of ROCKs blocks cytoskeletal remodeling and matrix gene expression in TGFβ stimulated fibroblasts [15,16,17], while ischemic hearts from ROCK1 knockout mice failed to form myofibroblasts and revealed less fibrosis [17] suggesting a critical role for the molecule during cardiac remodeling and scar formation.

In addition to the aforementioned key players, actin assembly can be influenced by numerous additional molecules and pathways within the cell. Thymosin beta-4 (TB4) is a 43 amino acid peptide originally discovered by A. Goldstein and A. White in 1966 [18]. Later, it was introduced as a central role player in regulating actin dynamics [19,20]. TB4 has been implicated in modulating the availability of actin monomers by sequestering G-actin, however, overexpression of TB4 indicated an alternative function for the molecule [20,21]. Accordingly, TB4 not only sequesters G-actin but also regulates actin assembly, acting at both filament ends, unlike profilin [21].

The role of the peptide regarding organ regeneration has been studied and documented for a quite considerable length of time. The first report highlighting the presence of TB4 in wound fluid by Frohm et al. was followed by numerous investigations supporting the role of endogenous TB4 in fighting fibrotic events and mechanisms in the body [22,23,24]. In addition to all benefits of endogenously increased TB4 expression, exogenous application of the molecule proved equally supportive in post-traumatic regenerative processes of various organs such as the brain [25,26,27], kidney [28,29,30], lung [31,32], eye [33,34,35], middle ear [36] or liver [37,38]. Our earlier research revealed TB4 plays an equally critical role in various pathological aspects of the heart [6,9,39]. We discovered the peptide is expressed during embryonic development [9] and is also capable of reminding the adult heart of its embryonic state by activating signaling mechanisms characteristic of the embryo [6,39,40,41,42]. Following acute hypoxia, systemic injection of the peptide increases heart function by inhibiting cell death, increasing vascular growth and significantly reducing fibrotic scarring in adult mice and pigs [9,40]. These results were equally supported by numerous investigations suggesting a potential for TB4 to treat humans with heart disease [24,39,43,44,45,46,47,48,49,50,51,52].

One of the major advantages of TB4 in tissue repair is its ability to reduce rigid, malfunctioning scar tissue, likely achieved through the suppression of inflammatory processes [53,54,55], inhibition of cellular death by activating Akt [9] and by general promotion of additional repair mechanisms [6,22,24,56,57,58]. Notably, the peptide’s anti-fibrotic capacities were strongly interconnected to the first four amino acids of its N-terminal domain, Ac-SDKP [24].

Although the molecular mechanisms triggered by TB4 during the various repair processes were widely investigated [22,24], many questions remain unanswered regarding its role in post-ischemic tissues. The aim of this present study was to reveal further targets and novel mechanisms through which TB4 mitigates pathological scarring in the heart. Accordingly, we delved into the molecular alterations through which TB4 may influence scar formation in vivo following hypoxia.

## 2. Results

### 2.1. TB4 Alters miR139-5p Expression in Adult Infarcted Mammalian Hearts

**Figure 1:**
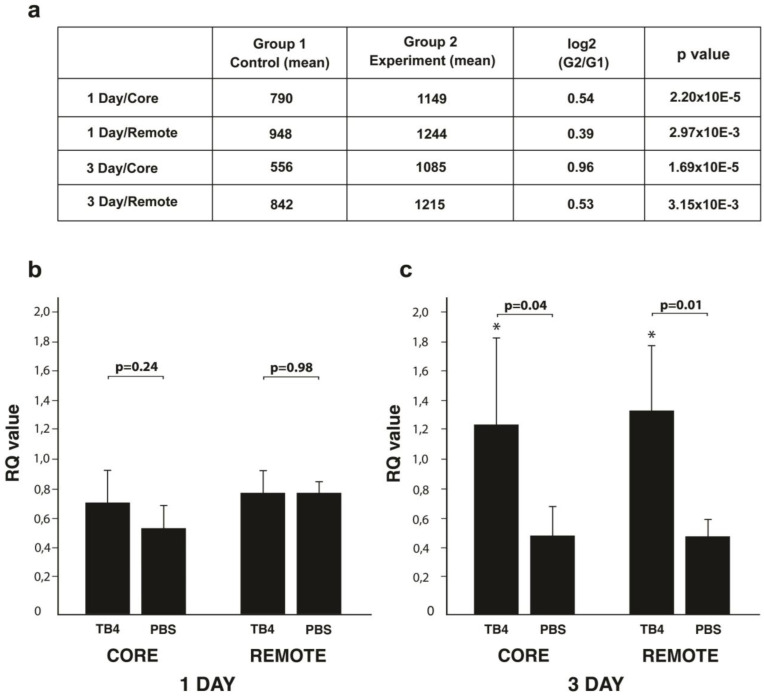
RT-PCR confirmed miR139-5p upregulation in response to TB4 treatment in the hypoxic mammalian heart. (**a**) MicroRNA microarray screens indicate a significant increase in miR139-5p expression in response to TB4 treatment one and three days following coronary artery ligation in the infarcted core (*n* = 4 for each treatment/time point/area, respectively). (**b**,**c**) Real-time PCR investigations revealed a significant increase in miR139-5p expression in the remote areas three days following systemic TB4 administration (**c**). We did not detect significant alterations on the first day of treatment (**b**). Bars indicate standard deviation at 95% confidence intervals (RFU: Relative Fluorescence Unit; *n* = 4 treatment/time point/area, * *p* < 0.05).

Our previous results suggested reduction of cellular death is one, but most likely not the only mechanism responsible for TB4′s positive impact following hypoxic events in the heart [9]. To further elucidate the potential molecular mechanisms beyond our observations, we performed microRNA microarrays one and three days following infarction, with or without TB4 treatment, utilizing samples from the core and remote areas of adult mouse hearts (*n* = 4 core/remote, one day/three days/each, respectively). In addition to numerous altered potential targets, we discovered miR139-5p became significantly elevated one and three days following systemic treatment with TB4 when compared to PBS-treated controls (Figure 1a).

To confirm microarray results, we performed real-time PCR utilizing miR139-5p specific probe on the core and remote areas of the hearts (*n* = 4/each). We observed a slight but not yet significant increase in the infarcted core, but none in the remote areas of the hearts one day following infarction (RQ values/core: TB4, 0.708 +/− 0.219, PBS, 0.536 +/− 0.146, 1.32-fold change, *p* = 0.24; RQ values/remote: TB4, 0.787 +/− 0.146, PBS, 0.786 +/− 0.066, 1.01-fold change *p* = 0.98, *n* = 4/each) (Figure 1b). Our results however indicated a significant upregulation in miR139-5p expression on the third day in both the core and remote areas of the hearts when compared to PBS treated controls (RQ values/core: TB4, 1.262 +/− 0.569, PBS, 0.536 +/− 0.188, 2.25-fold change, *p* = 0.04; RQ values/remote: TB4, 1.210 +/− 0.379, PBS, 0.466 +/− 0.095, 2.59-fold change *p* = 0.01, *n* = 4/each) (Figure 1c).

### 2.2. miR139-5p Target ROCK1 Decreases Following TB4 Treatment in Adult Infarcted Mammalian Hearts

**Figure 2:**
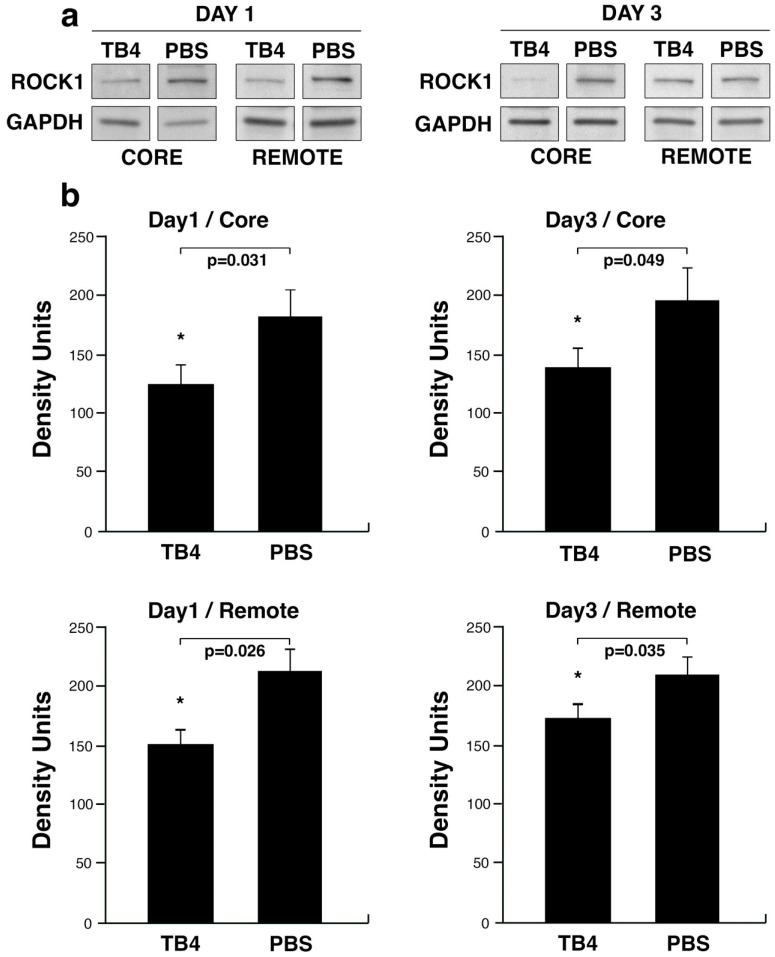
TB4 downregulates ROCK1 protein levels in vivo following cardiac infarction. (**a**) Representative western blot images of the core and healthy remote areas utilizing ROCK1 primary antibody. Our analyses revealed ROCK1 to be significantly decreased at days one and three in the infarcted core and remote areas of the hypoxic adult mouse heart following systemic TB4 treatment when compared to PBS. (**b**) Bar charts represent the calculated density units normalized to the total protein input by Coomassie stain. Bars indicate standard deviation at 95% confidence intervals (*n* = 3/each, * *p* < 0.05).

Next, we searched for predicted potential targets of miR139-5p utilizing Targetscan (targetscan.org) software in parallel with the literature. Our attention was primarily focused on molecules which are expressed in the heart and described as critical during the processes of remodeling and fibrotic scar formation. One potential target of miR139-5p that meets these criteria and was previously identified by Zhao et al. is ROCK1 [59], a well-known regulator of actin assembly in various cell types [60]. To reveal whether TB4 may influence ROCK1 protein expression in general, we first performed western blot analyses utilizing protein samples from the core and remote uninjured areas of mouse hearts one and three days following infarction and simultaneous systemic TB4 or PBS treatment. The source for protein samples was the same tissue used for microarray analyses. Our results demonstrated TB4 significantly decreases ROCK1 protein levels in the core and remote areas of the heart one and three days following cardiac infarction respectively (Density units/1 day core: TB4, 124.52+/−17.2, PBS, 182.37+/−23.70, *p* = 0.031; Density units/1 day remote: TB4, 151.34+/−13.15, PBS, 213.27+/−23.45, *p* = 0.026; Density units/3 day core: TB4, 139.25+/−16.63, PBS, 196.10+/−27.74, *p* = 0.049; Density units/3 day remote: TB4, 173.43+/−11.63, PBS, 209.12+/−15.16, *p* = 0.035 *n* = 3/each, respectively) (Figure 2). Contrary to the findings with ROCK1, western blots with ROCK2-specific primary antibody did not reveal significant alterations of the protein following systemic TB4 treatment in infarcted mouse hearts (Figure S1).

### 2.3. ROCK1 mRNA Expression Following Infarction

**Figure 3:**
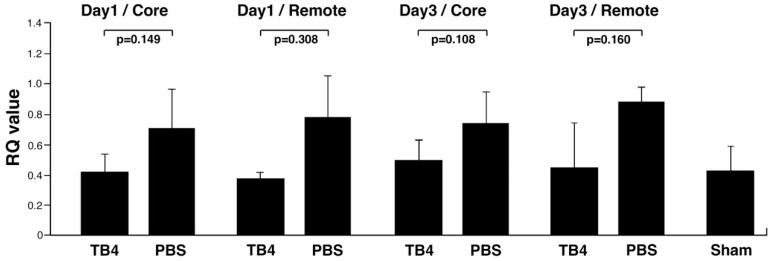
mRNA expression of ROCK1 in TB4-treated mouse hearts. Real-time mRNA expression analyses of ROCK1 revealed a decrease in mRNA levels in the infarcted core and remote areas following one and three days of systemic TB4 treatment in the hypoxic hearts when compared to PBS-treated and sham-operated controls. Bars indicate standard deviation at 95% confidence intervals (*n* = 3/each) (All measurements were performed in triplicates).

Our results indicate ROCK1 protein levels dropped, however, miR139-5p remained unaltered on day one following infarction. To search for additional potential mechanisms responsible for ROCK1 alterations, we investigated whether ROCK1 mRNA levels did alter at these time points in the heart. Our results indicated ROCK1 mRNA decreases visibly yet not significantly in all remote and healthy tissues of the infarcted mouse hearts during the first 24 h (RQ value: Day1 Core: TB4, 0.426 +/− 0.107, PBS, 0.712 +/− 0.256 *p* = 0.149; Day1 Remote: TB4, 0.374 +/− 0.045, PBS, 0.781 +/− 0.281 *p* = 0.308) and three days (RQ value: Day3 Core: TB4, 0.502 +/− 0.135, PBS, 0.741 +/− 0.214 *p* = 0.108; Day3 Remote: TB4, 0.452 +/− 0.286, PBS, 0.889 +/− 0.097 *p* = 0.160) following systemic TB4 treatment at the core and remote regions (Figure 3), respectively. Notably, the RQ values of TB4 of all regions and treatments were close or similar to those detected in the sham-operated hearts (RQ value: 0.435 +/− 0.159).

### 2.4. TB4 Decreases ROCK1 Protein Levels in the Hypoxic Adult Mammalian Mouse Heart In Vivo—Immunohistochemistry

**Figure 4:**
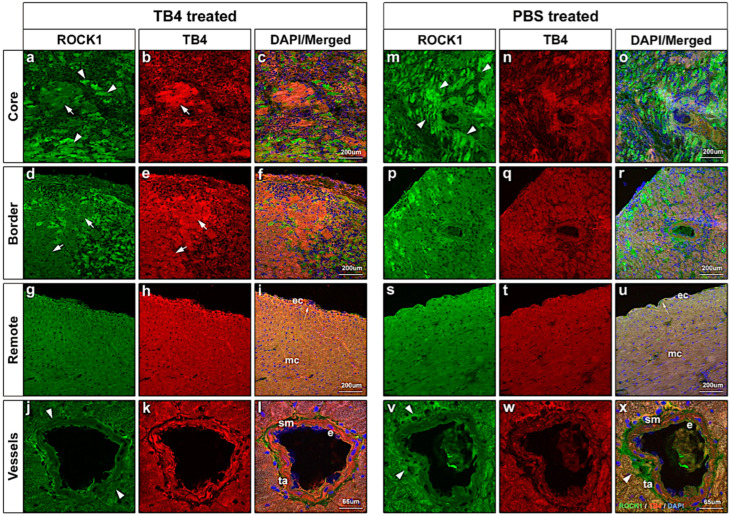
Immunohistochemical analyses of infarcted mouse hearts three days following MI. ROCK1 protein levels decreased in response to TB4 treatment at the infarcted core (**a**–**c**), border (**d**–**f**) and remote (**g**–**i**) areas when compared to PBS-treated controls (**m**–**u**). At (**a**,**m**), arrowheads indicate ROCK1-positive cardiac muscle cells. At (**a**,**b**,**d**,**e**), white arrows point to surviving cardiomyocytes with strong TB4 positivity and low ROCK1 expression in the TB4-treated infarcted core and border regions. In panel (**i**) a thickening of the epicardial layer (white arrow) may be observed when compared to PBS-treated hearts (**u**), indicating activation of local progenitors following TB4 treatment. (**g**–**j**,**s**–**u**) Immunohistological analysis indicates increased ROCK1 positivity in the remote areas (**g**) when compared to PBS-treated controls (**s**). (**j**–**l**,**v**–**x**) High-powered microscopic images reveal a decrease of ROCK1 protein levels in the tunica adventitia of the mature cardiac vessels ((**j**,**v**,**x**) white arrows). Blue DAPI stain represents the nuclei of cells (e: vessel endothel; ta: tunica adventitia, sm: smooth muscle) (*n* = 3/each treatment).

To better understand and to identify the localization and type of cells in which ROCK1 protein expression becomes altered, we performed immunohistochemistry on cryo-preserved post-hypoxic mouse hearts. Supporting our western blot results, immunostaining revealed significant distinctions between TB4- and PBS-treated hearts (Figure 4). First, in alignment with our earlier findings [9], we re-confirmed systematic TB4 injection results in a detectable increase in TB4 levels in all (core, remote and border) areas of the heart (Figure 4b,e,h,n,q,t). Next, correspondingly to western blots (Figure 2), ROCK1 was equally decreased in the core, border and remote regions following TB4 treatment. Finally, in the core and border areas, we found cardiac myocytes with increased TB4 positivity reveal a decrease in ROCK1 protein levels (Figure 4a,b,d,e white arrows) in comparison to those with low TB4. In the remote, non-infarcted areas, ROCK1 was decreased in the TB4-treated hearts (Figure 4g–i) when compared to those with PBS injection (Figure 4s–u). Lastly, we investigated TB4-initiated ROCK1 alterations in the mature cardiac vessels of the hearts (Figure 4j–l,v–x). The most significant differences were detectable in the outermost layer of the vessels (tunica adventitia (ta)), where the presence of ROCK1 was visibly increased in the hypoxic non-treated hearts (Figure 4j,v,x white arrowheads). TB4 expression in the smooth muscle cell layer (sm) was increased, but ROCK1 remained low in both TB4 and PBS-treated mature heart vessels. Finally, although the endothelial layer (e) was visibly positive for ROCK1, we did not detect significant alterations in this cell type by histochemistry.

### 2.5. In Vitro Effect of TB4 on ROCK1, Actin and MRTFA Levels in Adult Human Cardiac Cells

Since our immunohistochemical findings indicate TB4 alters ROCK1 levels in endothelial, myocardial and fibroblast cells of hypoxic mouse hearts, we asked whether these effects can be equally detected in human cardiac cells in vitro. Moreover, to elucidate the downstream effect of TB4-initiated ROCK1 alterations, we investigated the expression of actin and myocardin related transcription factor-A (MRTFA) [61,62] in all mentioned cell types, respectively.

**Figure 5:**
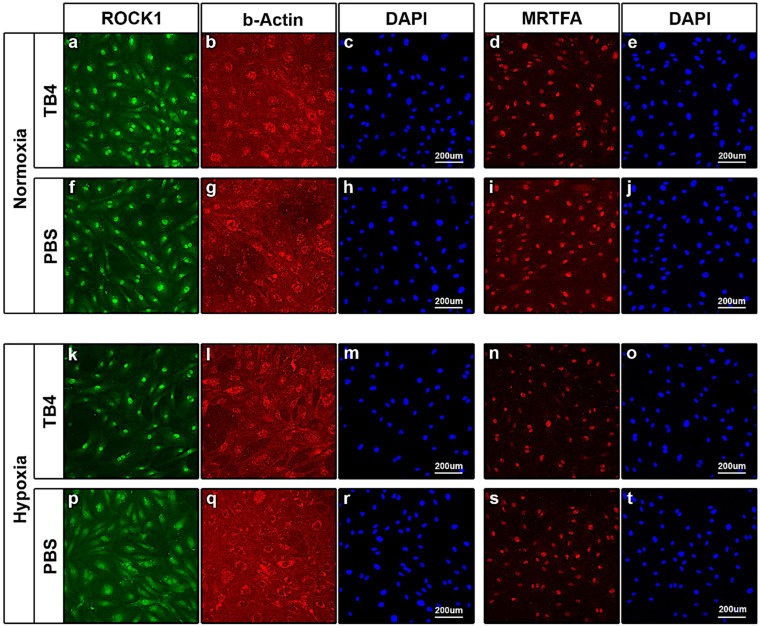
Alterations of ROCK1, beta-Actin and MRTFA proteins following external TB4 treatment of HUVECs in norm- and hypoxic conditions in vitro. (**a**–**j**) Immunocytochemical analyses detected no significant alterations in ROCK1 (**a**,**f**) and MRTFA (**d**,**i**) levels and localization following TB4 treatment with only a moderate disassembly of actin filaments when stained for beta-Actin (**b**,**g**) during normoxic conditions. (**k**–**t**) Under hypoxic conditions, immunocytochemistry revealed altered ROCK1 levels (**k**,**p**) and a visible drop in actin filaments (**l**,**q**) without detectable alterations in MRTFA levels and localization (**n**,**s**) following TB4 treatment when compared to PBS. Nuclei of cells stained with DAPI (blue) (**c**,**e**,**h**,**j**,**m**,**o**,**r**,**t**) (*n* = 3/each).

First, we treated human *umbilical vein endothelial cells (HUVECs)* with TB4 and PBS as control under normoxic and hypoxic conditions. Although our results indicated no significant alterations in ROCK1 expression under normoxic conditions by TB4 (Figure 5a,f), we detected a slight shift towards actin filament assembly (Figure 5b,g) when TB4 was not present in the culturing medium. During hypoxia, our results revealed TB4 decreases ROCK1 levels in HUVECs (Figure 5k,p). This impact was underscored by a visible shifting towards actin disassembly in the cells (Figure 5l,q). TB4 was formerly described to impact translocation towards the nuclei of cells, and thus activation of MRTFA [63], a transcription factor knowingly interacting with G-actin and critical in activating profibrotic genes with SRF [61,62]. Therefore, we investigated the impact of TB4 on MRTFA expression and translocation in HUVECs. Our result revealed no alterations in MRTFA localization as it was exclusively present in the nuclei of the cells during both hypoxic and normoxic conditions (Figure 5d,i,n,s).

**Figure 6:**
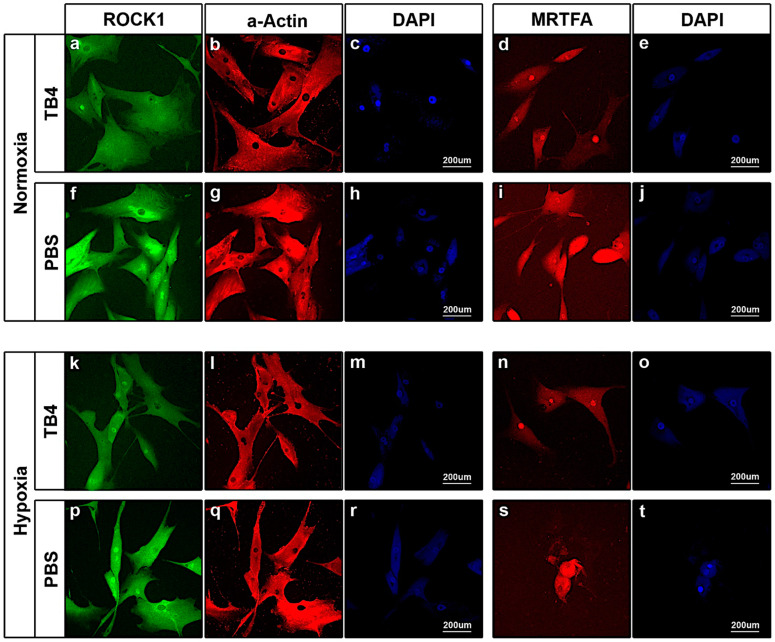
Alterations in ROCK1, alpha-Actin and MRTFA protein levels following external TB4 treatment of human adult CMs in norm- and hypoxic conditions in vitro. Immunocytochemistry of normoxic (**a**–**j**) and hypoxic (**k**–**t**) adult human heart cells revealed a significant decrease in ROCK1 protein levels with a decrease in filamentous actin structures following external TB4 treatment (**b**,**l**) when compared to those cells with PBS (**g**,**q**). (**d**,**i**,**n**,**s**) Indicate a localization shift of MRTF-A in hCMs towards the nucleus following TB4 addition under both hypoxic and normoxic conditions (**d**,**n**) when compared to PBS-treated cells (**i**,**s**), respectively. Nuclei were stained with DAPI (blue) (**c**,**e**,**h**,**j**,**m**,**o**,**r**,**t**) (*n* = 3/each).

Next, we asked how TB4 influences the behavior of the enlisted molecules in *adult human cardiac myocytes (hCMs)* (Figure 6). Much like our findings in adult mouse hearts (Figure 4), immunostaining of hCMs revealed a visible decrease in ROCK1 levels in both norm- or hypoxic conditions (Figure 6a,f,k,p), which phenomenon was underscored by a decrease in actin filament structures in TB4 treated cells (Figure 6b,l) when compared to PBS controls (Figure 6g,q). Moreover, in contrast to HUVECs, a significant shift of MRTFA towards the nucleus was unequivocally detectable in TB4-treated hCMs (Figure 6d,n), suggesting the previously described TB4-initiated MRTFA activation [61] is likely myocyte specific, at least in the human heart.

**Figure 7:**
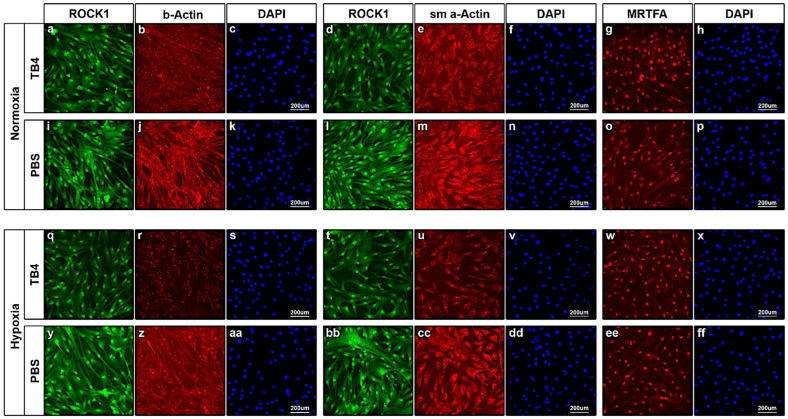
Alterations in ROCK1, alpha-Actin, alpha-smooth muscle Actin and MRTFA proteins of cultured human adult CFBs under normoxic and hypoxic conditions following TB4 treatment. Immunocytochemistry revealed a significant decrease in ROCK1 expression following TB4 treatment under both normoxic (**a**,**i**,**d**,**l**) and hypoxic (**q**,**y**,**t**,**bb**) conditions. (**b**,**j**,**r**,**z**) Panels demonstrate beta-actin filaments become disrupted following TB4 addition to the medium (**b**,**r**), and this alteration became significantly prominent under hypoxic conditions (**r**) when compared to PBS-treated cells (**j**,**z**). (**e**,**m**,**u**,**cc**) Panels reveal a detectable decrease in alpha-smooth muscle Actin positivity in TB4-treated hCFBs (**e**,**u**), especially under hypoxic conditions (**u**) when compared to PBS-treated cells (**m**,**cc**), suggesting sufficient inhibition of fibroblast-myofibroblast transformation in vitro. (**g**,**o**,**w**,**ee**) Panels show no significant alterations in MRTFA levels and localization following TB4 treatment (**g**,**w**) when compared to PBS (**o**,**ee**). Nuclei were stained with DAPI (blue) (**c**,**f**,**h**,**k**,**n**,**p**,**s**,**v**,**x**,**aa**,**dd**,**ff**) (*n* = 3/each).

Finally, we examined the impact of TB4 on *adult human cardiac fibroblasts* (hCFBs). In addition to the previously enlisted molecules, we investigated the alterations of alpha-smooth muscle Actin (sm a-Actin), a respected marker of fibroblast-myofibroblast transformation in the heart [15,64]. Our results revealed a substantial decrease in ROCK1 protein levels following TB4 treatment (Figure 7a,d,i,l,q,t,y,bb), which was most significant under hypoxic conditions (Figure 7q,t). Our observations regarding ROCK1 alterations were further supported by the proportional diminishing of filamentous actin structures in TB4-treated hCFBs, especially during hypoxia (Figure 7b,j,r,z). By investigating sm a-Actin, we found the levels of the protein decreased under both hypoxic and normoxic conditions by TB4 (Figure 7e,m,u,cc). Notably, TB4′s inhibition of fibroblast to myofibroblast transformation was most striking in hCFBs with hypoxia (Figure 7u,cc). Finally, in contrast to hCMs, we did not detect visible alterations in MRTFA localization by TB4 in this cell type, as MRTFA remained primarily restricted to the nucleus of hCFBs (Figure 7g,o,w,ee).

## 3. Discussion

The array of cardiovascular diseases and dysfunctions represent some of the most extensively studied health conditions and remain a leading cause of mortality worldwide. Stemming from a multifaceted background, the development of inflammation and its often consequential scarring contribute to malfunctions and decreased performance of the heart. Therefore, influencing distinct molecular mechanisms to reduce pathological scar formation through various agents holds significant clinical relevance and potential.

In our earlier studies we demonstrated TB4, a 43 amino acid peptide, is capable of enhancing cardiac function in adult mice and pigs [9,40,57]. In addition to inhibition of cell death and activation of vessel growth, functionality was restored and supported by a significant reduction in scar volume in both species [40,57]. To understand how TB4 may achieve these alterations, we performed miRNA microarrays while focusing on targets potentially influencing proteins related to cardiac remodeling. Accordingly, in this study, we identified a novel mechanism by which TB4 may mitigate scar formation following hypoxia in the heart. We found the peptide may indirectly (Figure 1) or directly (Figure 3) modulate ROCK1 protein levels in the adult mouse heart following infarction in vivo (Figure 2 and Figure 4). The inhibitory action was most evident in the core and border regions, where TB4-positive cells or cell islands exhibited visibly lower ROCK1 levels (Figure 4a,b,d,e white arrows). Moreover, ROCK1 became downregulated in both infarcted core and remote regions, suggesting the effect is not exclusively hypoxia dependent (Figure 2). In mature vessels, we identified an involvement of endothelial cells and cells of the adventitial layer, which includes fibroblasts and stem/progenitors within a collagen-rich connective tissue matrix [65]. Knowingly, adventitia also contains components with a critical role in immune surveillance and inflammatory cell trafficking [65] thus making our observations particularly intriguing when inhibition of scarring and pathological remodeling processes becomes a focal point of investigation. The lowering effect was equally detectable when utilizing human cardiac cells in vitro (Figure 5, Figure 6 and Figure 7).

Results of microRNA microarray and real-time PCR suggested upregulation of mir139-5p may be one of the mechanisms by which TB4 influences ROCK1 levels in the heart [59]. The precise mechanisms of TB4′s direct impact on ROCK1 mRNA expression however remain still unclear and are currently under investigation. Moreover, because of the relatively small sample size of our study, future research with an increased sample number will be equally important to fully validate our findings to ensure reproducibility and generalizability in a clinical setting.

The significance and role of ROCK1 have been investigated in the context of cardiac remodeling and consequential scar formation by many investigators [17,66,67,68]. The protein plays a crucial role in actin dynamics, which is essential for regulating various aspects of cellular physiology, including cell migration, proliferation, neurite extension and vesicle trafficking [69]. These processes are closely linked to actin polymerization and its association with stress fibers within cells [14]. These molecular assemblies are anchored to the plasma membrane through their association with focal adhesions and are responsible for generating contractile force via myosin-mediated anti-parallel motion of F-Actin. Reports show that inhibition of ROCK1 expression resulted in a decrease of stress fiber formation and focal adhesion assembly [70,71], while overexpression of the protein enhanced the number of fibers, thus contraction [69,70]. Consistent with knockout or inhibitory observations, in our present investigation we detected a visible decrease in actin filaments, especially in human cardiac fibroblasts and myocytes (Figure 6 and Figure 7).

Fibroblasts are tightly related to scar formation, not only as key sources for the extracellular matrix (ECM) of the healthy but also in the ischemic heart, where the significant loss of cardiomyocytes becomes replaced by cardiac fibroblasts, which then can differentiate into myofibroblasts [17,66,72,73,74]. In these cells, the expression of contractile proteins such as sm a-Actin becomes up-regulated together with an increased expression and secretion of MMPs and collagen fibrils [75] which eventually leads to the development of cardiac fibrosis. To alter these pathological and functionally unbeneficial processes, influencing ROCK1 activity was widely suggested. The findings and potential mechanisms were described by utilizing various in vivo and in vitro models, including modern 3D environments [66,67,68,73,76,77,78,79]. In our present results, the external administration of TB4 to human cardiac fibroblasts revealed a significant decrease of sm a-Actin not only during hypoxia but also under normoxic conditions (Figure 7e,u). This suggests a strong influence on the aforementioned critical transformation and thus on the consequential cellular alterations and pathologies in humans. Our results by immunocytochemistry suggest TB4 may achieve this phenomenon by decreasing ROCK1 protein levels in various cell types (Figure 7a,q). Regarding the heart, our results indicate TB4-initiated alterations are strongly cell type specific, which observation can be equally underscored by our earlier results showing altered cellular uptake of the C-terminal domain of TB4 into human cardiac endothelial cells and myocytes [57]. As presently demonstrated, in HUVECs, ROCK1 alterations were primarily detectable under hypoxic conditions (Figure 5k,p), while in cardiac myocytes and fibroblasts, TB4′s influence became visible under both hypoxic and normoxic conditions (Figure 6 and Figure 7). Moreover, the structural alterations of actin filaments were faithfully following the shift in ROCK1 levels in all cell types (Figure 5, Figure 6 and Figure 7 and Table S1). The question remains whether these alterations are mediated similarly or by a differing mechanism in ROCK1-expressing cells of the heart.

Earlier investigations were tightly interconnecting fibroblast maturation and vessel development with the actin-dependent Rho/MRTF/SRF pathways [16,79,80,81,82]. As suggested, by activation of Rho/ROCK signaling, polymerization of globular actin becomes initiated and a consequential release of MRTFs from their actin-bound state leads to MRTF translocation to the nucleus. There, it induces the expression of various profibrotic genes through the activation of SRF [83]. Recently, numerous studies have demonstrated that inhibition of the ROCK/MRTF/SRF signaling prevents myofibroblast activation and also promotes myofibroblast apoptosis, thus limiting the development of fibrosis in various organs, including the heart or the lung [68,79,84,85]. Moreover, a potential connection between TB4 and MRTF/SRF signaling has been equally reported, suggesting that an increase of TB4 in the cytosol initiates MRTF’s nuclear translocation by competitively sequestering G-actin and thus, releasing MRTF-A from its G-actin binding [61,62]. Our present findings however suggest TB4 initiated translocation of MRTF-A is strongly cell type specific (Figure 5, Figure 6 and Figure 7). While external addition of the peptide to human adult cardiac myocytes accelerated MRTF-A translocation to the nucleus under both hypoxic and normoxic conditions (Figure 6d,n vs. Figure 6i,s), we did not detect a similar transition in HUVECs (Figure 5d,n vs. Figure 5i,s) or in cardiac fibroblasts (Figure 7g,w vs. Figure 7o,ee, and Table S1). On the contrary, in these cells, we found MRTF-A was primarily located in the nucleus independently of TB4 addition, while ROCK1 levels were still decreased in each cell type. This suggests the detected decrease of sm a-Actin in TB4-treated adult fibroblasts may occur through MRTF-A-independent mechanisms in these cells.

Recent studies have shown that matrix stiffness, composition, cell adhesion, membrane tension and remodeling are all connected through several key pathways—such as FAK/Src, PI3K/Akt, and RhoA/ROCK—and may be regulated by shared molecular mechanisms [86]. Importantly, TB4 has been demonstrated to be influential regarding at least the first two pathways by our research team and others [9,87] as it became evident by this study that the peptide has a critical impact on ROCK1 expression and regulation, respectively. By observing the discrepancies between ROCK1 expression, sm a-Actin expression and MRTF-A’s altered behavior in human fibroblast and endothelial cells in comparison to cardiac myocytes, further investigation of TB4′s effects on downstream signaling pathways may be critical and is a subject of our current research.

In addition to ROCK1, its isoform ROCK2 has also been recognized as a key player in heart pathology [66,68,69,77,78]. ROCK1 and ROCK2 are highly homologous, with a 65% identity in their amino acid sequence and 92% homology in their kinase domains [66,68]. Despite their strong resemblance, differences in subcellular distribution, target proteins, and upstream regulatory mechanisms across various cell types may however lead to distinct functions [77]. Accordingly, there is a recognized need for selective ROCK inhibitors, not only to enhance our understanding regarding the molecular mechanisms driven by the two kinases but also to selectively target the pathological processes associated with ROCK1 and ROCK2 in the damaged heart. Examples of often utilized ROCK inhibitors are Y-27632 and Fasudil, which target the molecules’ ATP-dependent kinase domains and are therefore capable of equally inhibiting both ROCK1 and ROCK2 activity [68,79,88,89]. However, at higher concentrations, both are known to inhibit additional kinases, such as PKA and PKC [89] making these commercially available inhibitors less specific for ROCKs when compared to other serine-threonine kinases in vivo. Moreover, they are equally incapable of differentiating between ROCK1 and ROCK2 [78]. Our results with TB4 however did not reveal significant downregulation of ROCK2 in the core and remote areas of the infarcted hearts by western blot (Figure S1). Moreover, TB4 knowingly activates rather than inhibits PKC [40], making the peptide suitable for future studies of ROCK inhibition. However, the non-significant slight lowering in ROCK2 levels at three days following TB4 treatment (Figure S1) requires further analyses to assess the potential impact of the peptide on ROCK2 activity in heart cells. It is also important to note that in earlier studies, most externally administered drugs such as Y-27632 or Fasudil were primarily influencing the activity of ROCKs without altering their protein levels [68,79,88,89]. In our settings with TB4, the reduced activity of ROCK1 was likely due to lowered mRNA production in both remote and core regions of the heart one and three days following infarction (Figure 3), which was most likely further regulated by a significant increase of miR139-5p three days after infarction (Figure 1). Since externally administered TB4 is capable of reaching the nucleus of cells [9], elucidating the potential mechanisms of TB4′s impact on ROCK1 mRNA transcription warrants further analyses.

## 4. Conclusions

Heart regeneration is a complex process aimed at restoring cardiac function following injury. Sadly, the adult mammalian heart possesses limited regenerative capacities. Consequential myocardial fibrosis contributes to pathological remodeling and heart failure [90,91,92]. To counter these unprofitable transformations, identification of the molecular triggers and networks responsible for the missing functionality is undoubtedly critical. Therapeutic agents targeting key profibrotic pathways, such as ROCK signaling, have shown promise in supporting tissue repair [68]. However, further research is undoubtedly needed to refine these approaches and to minimize or eliminate side effects. Our current findings on TB4′s impact on ROCK1 expression and its ability to reduce profibrotic activity reveal a novel mechanism by which the peptide influences actin dynamics, making the molecule a promising candidate for future clinical applications.

## 5. Materials and Methods

### 5.1. Animal Procedures

Myocardial infarctions were produced in C57BL/6J male mice at 16 weeks of age (25–30 g) by ligation of the left anterior descending (LAD) coronary artery as previously described [9]. All animal protocols were reviewed and approved by the University of Texas Southwestern Medical School Institutional Animal Care Advisory Committee and were in full compliance with the rules governing animal use as published by the NIH. Briefly, mice were sedated in an isoflurane chamber (5%) for 60 s until a self-designed coaxial mask was safely applied. The mask supplied continuous isoflurane (1.2%) and oxygen (98.0%) under positive pressure from a Harvard small animal respirator throughout the procedures to reach ~650 beats/min steady heart rate. Immediately following permanent ligation, mice were injected with 150 μg peptide in 300 μL of PBS or 300 μL PBS intraperitoneally as described [9]. We administered buprenorphine (0.05 mg/kg) for post-operative pain control. Hearts were removed one and three days following ligation and processed for further investigations. Tissue samples were taken from the necrotic core, border, and healthy remote areas of the infarcted hearts. Total RNA and protein were isolated from the tissues using Trizol Reagent (Invitrogen, Carlsbad, CA, USA), and some were frozen for subsequent cryostatic immunohistochemistry or following fixation in 4% paraformaldehyde (PFA) embedded in paraffin in an appropriate medium and manner.

### 5.2. miRNA Microarray

To perform miRNA microarrays, we isolated RNA from the core (area of risk) and remote areas of four TB4-treated and four PBS-treated hearts utilizing Trizol Reagent (Invitrogen, Carlsbad, CA, USA) by following the manufacturer’s protocol (Invitrogen Carlsbad, CA, USA). Microarray assay was performed by a service provider (LC Sciences, Houston, TX, USA). Shortly, all assays were initiated using 10 μg of total RNA, size fractionated by a YM-100 Microcon centrifugal filter (Millipore, Burlington, MA, USA). The isolated small RNAs (<300 nt) were 3′-extended via a poly(A) tail, using poly(A) polymerase. An oligonucleotide tag was then ligated to the poly(A) tail for later fluorescent dye staining; two different tags were utilized for two RNA samples in dual-sample experiments. Data were analyzed by subtracting the background and signals were normalized using a locally weighted regression (LOWESS) filter [93,94]. For two-color experiments, the ratio of the two sets of detected signals (log2 transformed, balanced) and *p* values of the *t*-test were calculated; differentially detected signals were those with <0.05 *p* values.

### 5.3. Real-Time Quantitative PCR—miRNA

Total murine heart RNA was extracted utilizing Trizol Reagent (Invitrogen, Carlsbad, CA, USA) in full accordance with the manufacturer’s protocol (Invitrogen, Carlsbad, CA, USA). Following RNA quantification, 50 ng of total RNA from core and remote areas was transcribed to cDNA via TaqMan microRNA Reverse Transcriptase Kit (Thermo Fisher Scientific, Waltham, MA, USA). Additionally, miRNA-specific probes (mmu-miR139-5p and RNU6B endogenous control) (Applied Biosystems/Thermo Fisher Scientific, Waltham, MA, USA) were purchased and tested using cDNA templates from day one and day three TB4 and PBS-treated hearts, respectively (*n* = 3/each). Tissue miRNA alterations were detected utilizing StepOne Plus Real-Time instrument (Applied Biosystems/Thermo Fisher Scientific, Waltham, MA, USA) under the following cycle conditions: 1 × 50 °C for 2 min, 1 × 95 °C for 20 s, 40 × 95 °C for 1 s, and 60 °C for 20 s. A relative quantitation assay was used to analyze changes in miRNA expression following TB4 treatment relative to RNU6B reference following the manufacturer’s protocol.

### 5.4. Real-Time Quantitative PCR—mRNA

32 ng of total RNA from the core and remote areas of TB4 and PBS-treated hearts were utilized to determine ROCK1 mRNA expression one and three days following cardiac infarction. First-strand cDNA was synthesized by using oligo dT SuperScript™ IV First-Strand Synthesis System (Invitrogen, Carlsbad, CA, USA, Cat. No.: 18,091,050) in full accordance with the manufacturer’s instructions. Real-time-PCR was carried out utilizing StepOne Plus Real-Time instrument (Applied Biosystems/Thermo Fisher Scientific, Waltham, MA, USA) using TaqMan™ ROCK1 assay (assay ID: mm00485745_m1 Thermo Fischer Scientific) and Polr2a assay (Mm00839502_m1 Thermo Fischer Scientific) as endogenous control under the following cycle conditions: 1 × 48 °C for 30 min, 1 × 95 °C for 10 min, 40 × 95 °C for 15 s, and 60 °C for 1 min. Tissue mRNA alterations were detected via StepOne Plus Real-Time instrument (Applied Biosystems/Thermo Fisher Scientific, Waltham, MA, USA). A relative quantitation assay was used to analyze changes in mRNA expression following TB4 treatment relative to Polr2a reference following the manufacturer’s protocol.

### 5.5. Western Blot

Twenty-four and seventy-two hours following systemic injection of TB4 or PBS, mice were sedated. Hearts were perfused with saline to remove blood, and the infarcted core tissue was removed. The remaining intact area of the hearts was separated, and both samples were placed in 1 mL of Trizol reagent (Invitrogen, Carlsbad, CA, USA), followed by an immediate freezing in liquid nitrogen. The protein fraction was isolated from the interphase of the Trizol purification as recommended by the manufacturer; 12 μg of the total protein was analyzed by Western blot. Shortly, following denaturation in Laemmli buffer for 10 min at 95 °C, proteins were separated utilizing precast 4–20% polyacrylamide gels (4–20% Mini-PROTEAN^®^ TGX™ Precast Protein Gels, BioRad, Hercules, CA, USA) using a maximum of 15 mA constant current/gel in standard 1× running buffer (25 mM Tris 250 mM glycine (electrophoresis grade) (pH8.3), 0.1% SDS). Following separation, protein samples were transferred to PVDF membrane (Merck, Darmstadt, Germany) using 120 mA constant current for 130 min at room temperature. Following protein transfer, membranes were blocked in 5% nonfat dry milk/1xTris-Buffered Saline (TBS) solution for one hour at room temperature. ROCK1 (Abcam, Cambridge, UK), ROCK2 (Santa Cruz, Dallas, TX, USA) antibodies (1:500 dilution) and GAPDH antibodies (1:10,000 dilution) (Abcam, Cambridge, UK) were incubated overnight at 4 °C under gentle shaking. Following four times washing with 2% milk, 0.5% Tween 20 in TBS (Washing buffer), HRP-conjugated secondary antibodies (1:7500 dilution for anti-mouse, sc2005; 1:10,000 dilution for anti-rabbit sc-2313; Santa Cruz, Dallas, TX, USA) were added at room temperature for one hour in TBS. Following three times rinsing in washing buffer and once in PBS, target proteins were detected via Luminol Reagent (Santa Cruz, Dallas, TX, USA) using GeneSys imaging system from Syngene (Cambridge, UK). Accurate loading was investigated by directly staining the transferred proteins with Coomassie Blue staining on the membrane.

### 5.6. Cell Culturing—Normoxic and Hypoxic Conditions

Adult human umbilical vein endothelial cells (HUVECs), adult human cardiac fibroblasts (hCFBs) and adult human cardiac myocytes (hCMs) (PromoCell GmbH, Heidelberg, Germany) were cultured on collagen-treated cover glasses (Rat tail collagen, Roche) and incubated in cell-specific culturing medium (supplemented by the distributor; PromoCell GmbH, Heidelberg, Germany) until 30–60% confluency was achieved. 2 μg TB4 in PBS (*n* = 4) or 20 μL PBS (*n* = 4) alone was directly added to 2 mL of the culturing medium for 24 h under regular tissue culture conditions (20% O_2,_ 5% CO_2_, 75% N_2_). To achieve hypoxic stress, an equal number of cells with similar treatment conditions were incubated for 36 h (HUVECs and hCFBs) and 24 h (hCMs) under hypoxic conditions (5%CO_2_, 1%O_2_, 94% N_2_). Following incubation, cell culture experiments were terminated utilizing 4% PFA for 10 min at room temperature, rinsed and stored in PBS at 4 °C until immunostaining was performed.

### 5.7. Immunocytochemistry

PFA-fixed HUVEC, hCM or hCFB-containing collagen-coated coverslips were washed in PBS. Following washing, cells were permeabilized with Permeabilize solution (10 mmol/L PIPES pH 6.8, 50 mmol/L NaCl, 0.5% Triton X-100, 300 mmol/L sucrose, and 3 mmol/L MgCl_2_) for 10 min and rinsed twice with PBS for 5 min each at room temperature, respectively. After blocking and rinsing steps, primary detection antibodies were utilized: ROCK1(rabbit) 1:600 (Invitrogen, Carlsbad, CA, USA); ROCK1(mouse) 1:200 (Santa Cruz, Dallas, TX, USA); MLK1 (MRTF-A) 1:200 (Invitrogen, Carlsbad, CA, USA); ROCK2 1:300 (Santa Cruz, Dallas, TX, USA); sm a-Actin 1:400 (Sigma, St. Louis, MO, USA); b-Actin 1:200 (Invitrogen, Carlsbad, CA, USA); TB4 1:400 (Abcam, Cambridge, UK). Control experiments without primary antibodies were conducted to rule out nonspecific labeling by secondary antibodies. Following rinsing and incubation with fluorophore-conjugated secondary antibodies (FITC-conjugated anti-mouse 1:200; Cy3-conjugated anti-mouse 1:200; Cy3-conjugated anti-rabbit 1:200 (Jackson Immuno Research Europe Ltd., Cambridge, UK), the cells were rinsed, nuclei were labeled by DAPI (1 uM) (Thermo Fisher Scientific, Waltham, MA, USA), and incubated with equilibration buffer (Slow fade antifade Kit, Invitrogen, Carlsbad, CA, USA, catalog No.: S2828) for 10 min at room temperature. Cell-containing coverslips were placed on a glass microscope slide, covered, and documented via Zeiss LSM-710 confocal microscopy (Zeiss, Oberkochen, Germany).

### 5.8. Immunohistochemistry—Cryopreserved Sections

Cryopreserved sections were thawed for 20 min at room temperature, followed by 10 min of fixation in ice-cold acetone. Next, slides were rinsed 3 × 5 min in TBS and blocked for 45 min in 5% heat-inactivated donkey serum. Following blocking, sections were incubated utilizing target-specific primary antibodies (ROCK1 1:50 (Abcam, Cambridge, UK) and TB4 1:200 (Abcam, Cambridge, UK) in 1% donkey serum in TBS in a wet chamber for two hours at room temperature. Control experiments without primary antibodies were conducted to rule out nonspecific labeling by secondary antibodies. Following three times rinsing with TBS, fluorochrome-conjugated secondary antibodies (FITC-conjugated anti-mouse, 1:100; Cy3-conjugated anti-rabbit 1:100, (Jackson Immuno Research Europe Ltd., Cambridge, UK)) were added and incubated for 45 min at room temperature. Following three washing steps in TBS for 5 min, nuclei were counterstained with DAPI (1 uM) (Thermo Fisher Scientific, Waltham, MA, USA), rinsed in TBS and protected by antifade reagent (Slow fade anti-fade Kit, Invitrogen, Carlsbad, CA, USA; S2828), covered via glass coverslips and documented by Zeiss LSM-710 confocal microscopy.

### 5.9. Statistical Analyses

For microRNA microarrays, statistical tests and clustering analyses were provided by LC Sciences as part of the miRNA microarray service. The signal values were derived by background subtraction and normalization. Detectable transcripts were subjected to data processing statistics and signal intensities were listed as average values of repeating spots. One-way ANOVA and *t*-test were utilized to determine significance. *p* < 0.05 was considered statistically significant.

RT-PCR results and quantification of western blots were processed utilizing Microsoft^®^ Excel 16.96 or GraphPad^®^ Prism 10.1.1 software. Quantitative results were expressed as means +/− SD. *p* < 0.05 was considered statistically significant, whereas *p* < 0.01 was highly significant.

## Data Availability

Dataset available on request from the authors.

## Supplementary Materials

The following supporting information can be downloaded at: https://www.mdpi.com/article/10.3390/ijms26094131/s1.
