# Supplementary material for: Thymosin Beta-4 Modulates Cardiac Remodeling by Regulating ROCK1 Expression in Adult Mammals
Source: Int J Mol Sci. 2025 Apr 26;26(9):4131. doi: 10.3390/ijms26094131 (PMC12072014; doi:10.3390/ijms26094131)
Supplement: Supplementary file 1 [file ijms-26-04131-s001.zip › ijms-3486734-supplementary.pdf]

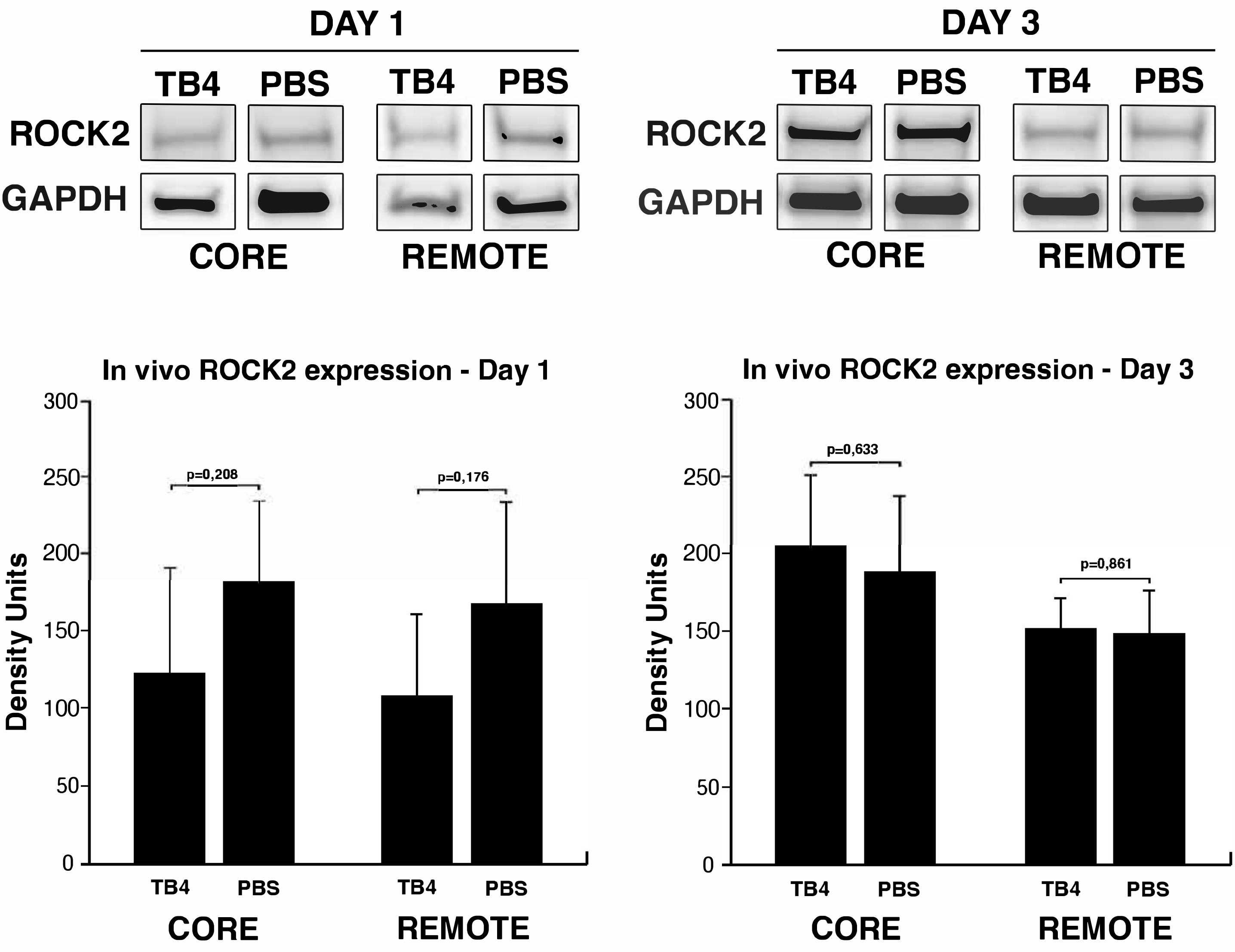

Supplementary Figure S1

TB4 does not alter ROCK2 protein levels in vivo following cardiac infarction. Western blot analysis revealed ROCK2 to be slightly but not significantly decreased at day one and unaltered at day three in the infarcted core and remote areas of the hypoxic adult mouse heart following systemic TB4 treatment. Bar charts represent the calculated density units normalized to GAPDH and total protein input by Coomassie stain. Bars indicate standard deviation at 95% confidence intervals (n=4, *<0.05).

Effects / TB4	**ROCK1**	**Filamentous-Actin assembly**	**MRTF-A translocation**	**sm a-Actin**
**Normoxia**				
HUVECs	Unaltered	Decreased+	Unaltered (nuclear)	N/A
hCMs	Decreased++	Decreased++	Nuclear translocation	N/A
hCFBs	Decreased+	Decreased++	Unaltered (nuclear)	Decreased+
**Hypoxia**				
HUVECs	Decreased++	Decreased++	Unaltered (nuclear)	N/A
hCMs	Decreased++	Decreased++	Nuclear translocation	N/A
hCFBs	Decreased++	Decreased+++	Unaltered (nuclear)	Decreased++

Supplementary Table S1. Alterations in ROCK1, Filamentous-Actin, MRTF-A and smooth muscle alpha-Actin expression in human cardiac cells following external addition of TB4 compared to PBS controls in vitro. HUVECs: human umbilical vein endothelial cells, hCMs: human cardiac myocytes, hCFBs: human cardiac fibroblasts. +: intensity level of the observed alteration
